# 2-Phenyl-1*H*-1,3,7,8-tetra­azacyclo­penta­[*l*]phenanthrene

**DOI:** 10.1107/S1600536808029759

**Published:** 2008-09-20

**Authors:** Hong-Min Xi

**Affiliations:** a Department of Chemistry, College of Chemistry and Biology, Beihua University, Jilin City 132013, People’s Republic of China

## Abstract

There are two molecules in the asymmetric unit of the title compound, C_19_H_12_N_4_. One is almost planar [dihedral angle between the fused-ring system and the phenyl ring = 2.16 (13)°] and one is somewhat twisted [dihedral angle = 13.30 (14)°]. In the crystal, the molecules are linked by N—H⋯N hydrogen bonds to result in chains.

## Related literature

For related literature, see Zhang *et al.* (2008 ▶); Yin (2008 ▶).
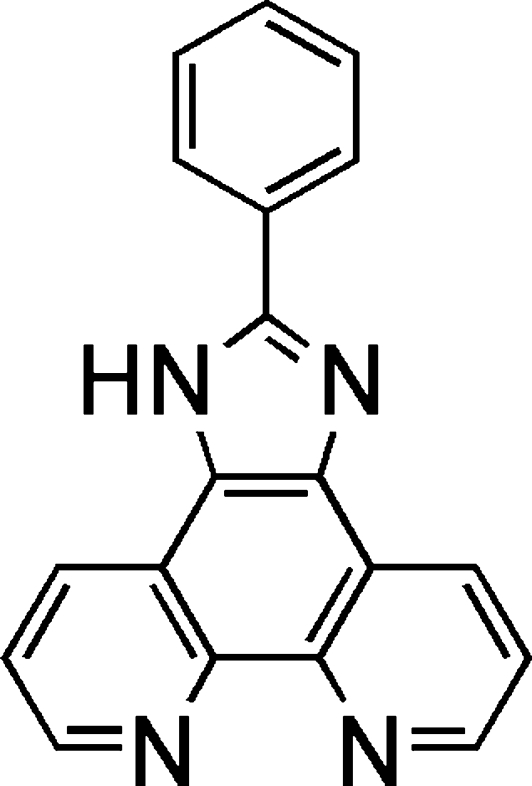


## Experimental

### 

#### Crystal data

C_19_H_12_N_4_
*M* *_r_* = 296.33
Monoclinic, 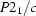

*a* = 12.3326 (15) Å
*b* = 12.2334 (15) Å
*c* = 19.885 (2) Å
β = 104.010 (2)°
*V* = 2910.9 (6) Å^3^
*Z* = 8
Mo *K*α radiation
μ = 0.08 mm^−1^
*T* = 293 (2) K
0.24 × 0.21 × 0.19 mm

#### Data collection

Bruker APEX CCD area-detector diffractometer
Absorption correction: multi-scan (*SADABS*; Bruker, 1998 ▶) *T*_min_ = 0.981, *T*_max_ = 0.982
23942 measured reflections
5721 independent reflections
2627 reflections with *I* > 2σ(*I*)
*R*_int_ = 0.089

#### Refinement

*R*[*F* ^2^ > 2σ(*F* ^2^)] = 0.060
*wR*(*F* ^2^) = 0.167
*S* = 0.97
5721 reflections
415 parameters
H-atom parameters constrained
Δρ_max_ = 0.61 e Å^−3^
Δρ_min_ = −0.26 e Å^−3^

### 

Data collection: *SMART* (Bruker, 1998 ▶); cell refinement: *SAINT* (Bruker, 1998 ▶); data reduction: *SAINT*; program(s) used to solve structure: *SHELXS97* (Sheldrick, 2008 ▶); program(s) used to refine structure: *SHELXL97* (Sheldrick, 2008 ▶); molecular graphics: *SHELXTL* (Sheldrick, 2008 ▶); software used to prepare material for publication: *SHELXTL*.

## Supplementary Material

Crystal structure: contains datablocks global, I. DOI: 10.1107/S1600536808029759/bt2790sup1.cif

Structure factors: contains datablocks I. DOI: 10.1107/S1600536808029759/bt2790Isup2.hkl

Additional supplementary materials:  crystallographic information; 3D view; checkCIF report

## Figures and Tables

**Table 1:** Hydrogen-bond geometry (Å, °)

*D*—H⋯*A*	*D*—H	H⋯*A*	*D*⋯*A*	*D*—H⋯*A*
N3—H3*A*⋯N6^i^	0.86	2.09	2.932 (4)	165
N8—H8*A*⋯N2^i^	0.86	2.12	2.948 (3)	163

Symmetry code: (i) 
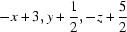
.

**Fig. 1.**
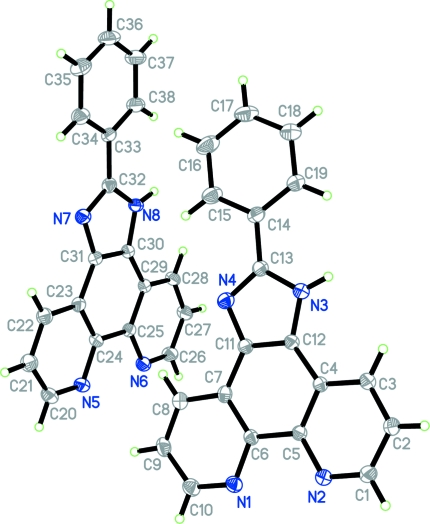
The structure of (I), showing the atomic numbering scheme. Displacement ellipsoids are drawn at the 30% probability level.

## supplementary crystallographic information

### Comment

1,10-Phenanthroline and its derivatives are widely utilized as ligands in metal complexes (Zhang *et al.*, 2008). I report here the crystal structure of the title compound, which was synthesized from 1,10-phenanthroline-5,6-dione. In this compound, all the bond lengths are within normal ranges (Yin, 2008). The asymmetric unit consists of two independent  molecules (Fig. 1), which are connected by N—H···N hydrogen bonds to form a one-dimensional chain (Table 1).

### Experimental

1,10-Phenanthroline-5,6-dione (1.5 mmol) and benzaldehyde (1.5 mmol) were dissolved in CH_3_COOHCH_3_COONH_4_ (1:1) solution (30 ml). The mixture was refluxed for 3 h under argon, after cooling, this mixture was diluted with water and neutralized with concentrated aqueous ammonia, immediately resulting a yellow precipitate, which was washed with water, acetone and diethyl ether respectively. Crystals of the title compound were obtained by recrystallization from dichloromethane.

### Refinement

C– and N-bound H atoms were positioned geometrically (N—H = 0.86 Å and C—H= 0.93 Å) and refined as riding, with *U*_iso_(H)= 1.2 *U*_eq_(carrier).

### Crystal data

**Table:**

C_19_H_12_N_4_	*F*(000) = 1232
*M**_r_* = 296.33	*D*_x_ = 1.352 Mg m^−^^3^
Monoclinic, *P*2_1_/*c*	Mo *K*α radiation, λ = 0.71073 Å
Hall symbol: -P 2ybc	Cell parameters from 5721 reflections
*a* = 12.3326 (15) Å	θ = 1.1–26.0°
*b* = 12.2334 (15) Å	µ = 0.08 mm^−^^1^
*c* = 19.885 (2) Å	*T* = 293 K
β = 104.010 (2)°	Block, pale yellow
*V* = 2910.9 (6) Å^3^	0.24 × 0.21 × 0.19 mm
*Z* = 8	

### Data collection

**Table:**

Bruker APEX CCD area-detector diffractometer	5721 independent reflections
Radiation source: fine-focus sealed tube	2627 reflections with *I* > 2σ(*I*)
graphite	*R*_int_ = 0.089
φ and ω scans	θ_max_ = 26.0°, θ_min_ = 1.7°
Absorption correction: multi-scan (SAINT; Bruker, 1998)	*h* = −15→15
*T*_min_ = 0.981, *T*_max_ = 0.982	*k* = −15→14
23942 measured reflections	*l* = −24→24

### Refinement

**Table:**

Refinement on *F*^2^	Primary atom site location: structure-invariant direct methods
Least-squares matrix: full	Secondary atom site location: difference Fourier map
*R*[*F*^2^ > 2σ(*F*^2^)] = 0.060	Hydrogen site location: inferred from neighbouring sites
*wR*(*F*^2^) = 0.167	H-atom parameters constrained
*S* = 0.97	*w* = 1/[σ^2^(*F*_o_^2^) + (0.0719*P*)^2^] where *P* = (*F*_o_^2^ + 2*F*_c_^2^)/3
5721 reflections	(Δ/σ)_max_ < 0.001
415 parameters	Δρ_max_ = 0.62 e Å^−^^3^
0 restraints	Δρ_min_ = −0.26 e Å^−^^3^

### Special details

**Table:**

Geometry. All e.s.d.'s (except the e.s.d. in the dihedral angle between two l.s. planes) are estimated using the full covariance matrix. The cell e.s.d.'s are taken into account individually in the estimation of e.s.d.'s in distances, angles and torsion angles; correlations between e.s.d.'s in cell parameters are only used when they are defined by crystal symmetry. An approximate (isotropic) treatment of cell e.s.d.'s is used for estimating e.s.d.'s involving l.s. planes.
Refinement. Refinement of *F*^2^ against ALL reflections. The weighted *R*-factor *wR* and goodness of fit *S* are based on *F*^2^, conventional *R*-factors *R* are based on *F*, with *F* set to zero for negative *F*^2^. The threshold expression of *F*^2^ > σ(*F*^2^) is used only for calculating *R*-factors(gt) *etc*. and is not relevant to the choice of reflections for refinement. *R*-factors based on *F*^2^ are statistically about twice as large as those based on *F*, and *R*- factors based on ALL data will be even larger.

### Fractional atomic coordinates and isotropic or equivalent isotropic displacement parameters (Å^2^)

**Table:**

	*x*	*y*	*z*	*U*_iso_*/*U*_eq_	
C1	1.8134 (3)	−0.7259 (3)	1.53329 (17)	0.0535 (9)	
H1	1.8713	−0.7628	1.5634	0.064*	
C2	1.7966 (3)	−0.6167 (2)	1.54683 (16)	0.0504 (8)	
H2	1.8415	−0.5822	1.5853	0.061*	
C3	1.7134 (2)	−0.5611 (2)	1.50287 (16)	0.0475 (8)	
H3	1.7017	−0.4875	1.5103	0.057*	
C4	1.6452 (2)	−0.6157 (2)	1.44615 (15)	0.0400 (7)	
C5	1.6667 (2)	−0.7273 (2)	1.43635 (15)	0.0399 (7)	
C6	1.5946 (2)	−0.7898 (2)	1.38033 (15)	0.0405 (7)	
C7	1.5008 (2)	−0.7402 (2)	1.33678 (15)	0.0423 (7)	
C8	1.4329 (3)	−0.8051 (3)	1.28464 (16)	0.0510 (8)	
H8	1.3714	−0.7748	1.2537	0.061*	
C9	1.4585 (3)	−0.9123 (3)	1.28015 (17)	0.0558 (9)	
H9	1.4135	−0.9571	1.2470	0.067*	
C10	1.5533 (3)	−0.9541 (3)	1.32599 (18)	0.0567 (9)	
H10	1.5704	−1.0274	1.3216	0.068*	
C11	1.4801 (2)	−0.6274 (2)	1.34767 (15)	0.0423 (7)	
C12	1.5524 (2)	−0.5691 (2)	1.39835 (16)	0.0417 (7)	
C13	1.4147 (3)	−0.4650 (2)	1.34191 (16)	0.0465 (8)	
C14	1.3452 (3)	−0.3683 (3)	1.32024 (17)	0.0509 (8)	
C15	1.2420 (3)	−0.3792 (3)	1.27505 (18)	0.0676 (10)	
H15	1.2177	−0.4474	1.2568	0.081*	
C16	1.1741 (3)	−0.2880 (4)	1.25679 (19)	0.0840 (13)	
H16	1.1044	−0.2959	1.2262	0.101*	
C17	1.2078 (4)	−0.1869 (3)	1.2829 (2)	0.0820 (12)	
H17	1.1615	−0.1266	1.2701	0.098*	
C18	1.3100 (4)	−0.1754 (3)	1.3277 (2)	0.0866 (13)	
H18	1.3334	−0.1070	1.3461	0.104*	
C19	1.3788 (3)	−0.2654 (3)	1.3460 (2)	0.0755 (12)	
H19	1.4488	−0.2567	1.3761	0.091*	
C20	1.1864 (3)	−0.9545 (3)	1.14281 (18)	0.0627 (10)	
H20	1.2040	−1.0282	1.1500	0.075*	
C21	1.1001 (3)	−0.9129 (3)	1.16926 (18)	0.0620 (10)	
H21	1.0615	−0.9575	1.1934	0.074*	
C22	1.0735 (3)	−0.8046 (3)	1.15866 (17)	0.0549 (9)	
H22	1.0172	−0.7741	1.1764	0.066*	
C23	1.1315 (2)	−0.7401 (2)	1.12107 (15)	0.0441 (8)	
C24	1.2181 (2)	−0.7894 (2)	1.09701 (15)	0.0425 (7)	
C25	1.2812 (2)	−0.7264 (2)	1.05692 (15)	0.0421 (7)	
C26	1.4170 (3)	−0.7220 (3)	0.99579 (17)	0.0548 (9)	
H26	1.4723	−0.7579	0.9797	0.066*	
C27	1.3965 (3)	−0.6129 (2)	0.97781 (17)	0.0540 (9)	
H27	1.4362	−0.5774	0.9500	0.065*	
C28	1.3169 (3)	−0.5593 (2)	1.00190 (16)	0.0486 (8)	
H28	1.3023	−0.4860	0.9912	0.058*	
C29	1.2573 (2)	−0.6147 (2)	1.04264 (15)	0.0409 (7)	
C30	1.1711 (2)	−0.5686 (2)	1.06952 (15)	0.0424 (8)	
C31	1.1082 (2)	−0.6270 (2)	1.10553 (15)	0.0429 (7)	
C32	1.0428 (2)	−0.4646 (3)	1.09756 (16)	0.0446 (8)	
C33	0.9771 (2)	−0.3676 (2)	1.10415 (16)	0.0458 (8)	
C34	0.8959 (3)	−0.3745 (3)	1.14149 (18)	0.0628 (10)	
H34	0.8819	−0.4411	1.1603	0.075*	
C35	0.8357 (3)	−0.2833 (3)	1.1510 (2)	0.0745 (11)	
H35	0.7819	−0.2887	1.1765	0.089*	
C36	0.8546 (3)	−0.1844 (3)	1.1231 (2)	0.0762 (12)	
H36	0.8143	−0.1228	1.1299	0.091*	
C37	0.9327 (3)	−0.1776 (3)	1.0856 (2)	0.0741 (11)	
H37	0.9449	−0.1111	1.0660	0.089*	
C38	0.9945 (3)	−0.2677 (3)	1.07615 (18)	0.0614 (9)	
H38	1.0482	−0.2612	1.0507	0.074*	
N1	1.6202 (2)	−0.8969 (2)	1.37510 (13)	0.0491 (7)	
N2	1.7522 (2)	−0.78066 (19)	1.48026 (13)	0.0470 (7)	
N3	1.5101 (2)	−0.46462 (19)	1.39421 (13)	0.0499 (7)	
H3A	1.5380	−0.4098	1.4196	0.060*	
N4	1.3927 (2)	−0.5633 (2)	1.31277 (13)	0.0501 (7)	
N5	1.2449 (2)	−0.8967 (2)	1.10833 (14)	0.0530 (7)	
N6	1.3630 (2)	−0.7781 (2)	1.03437 (13)	0.0504 (7)	
N7	1.0279 (2)	−0.5624 (2)	1.12287 (13)	0.0488 (7)	
N8	1.12765 (19)	−0.46471 (18)	1.06446 (12)	0.0439 (6)	
H8A	1.1498	−0.4099	1.0442	0.053*	

### Atomic displacement parameters (Å^2^)

**Table:**

	*U*^11^	*U*^22^	*U*^33^	*U*^12^	*U*^13^	*U*^23^
C1	0.051 (2)	0.051 (2)	0.053 (2)	0.0050 (17)	0.0021 (18)	−0.0024 (17)
C2	0.050 (2)	0.048 (2)	0.049 (2)	−0.0014 (17)	0.0046 (17)	−0.0064 (16)
C3	0.0459 (19)	0.0405 (18)	0.056 (2)	−0.0025 (15)	0.0121 (17)	−0.0003 (16)
C4	0.0371 (17)	0.0367 (18)	0.0460 (19)	−0.0029 (14)	0.0099 (15)	0.0026 (15)
C5	0.0350 (17)	0.0377 (17)	0.0485 (19)	0.0002 (14)	0.0130 (15)	0.0009 (15)
C6	0.0411 (18)	0.0381 (18)	0.0437 (19)	−0.0043 (14)	0.0133 (16)	−0.0010 (14)
C7	0.0394 (18)	0.049 (2)	0.0398 (19)	−0.0057 (15)	0.0126 (15)	−0.0012 (15)
C8	0.048 (2)	0.052 (2)	0.052 (2)	−0.0050 (16)	0.0106 (17)	−0.0021 (17)
C9	0.054 (2)	0.054 (2)	0.058 (2)	−0.0138 (17)	0.0112 (19)	−0.0162 (17)
C10	0.062 (2)	0.048 (2)	0.061 (2)	−0.0018 (18)	0.016 (2)	−0.0136 (17)
C11	0.0397 (18)	0.0403 (18)	0.048 (2)	−0.0015 (15)	0.0126 (16)	0.0049 (15)
C12	0.0385 (17)	0.0366 (18)	0.050 (2)	0.0002 (15)	0.0111 (16)	0.0039 (15)
C13	0.0422 (19)	0.045 (2)	0.052 (2)	0.0008 (16)	0.0107 (17)	0.0044 (16)
C14	0.044 (2)	0.053 (2)	0.056 (2)	0.0071 (17)	0.0129 (17)	0.0060 (17)
C15	0.063 (2)	0.073 (3)	0.059 (2)	0.018 (2)	0.000 (2)	−0.008 (2)
C16	0.068 (3)	0.101 (3)	0.070 (3)	0.032 (3)	−0.007 (2)	−0.004 (3)
C17	0.083 (3)	0.070 (3)	0.088 (3)	0.033 (2)	0.011 (3)	0.014 (2)
C18	0.075 (3)	0.055 (3)	0.121 (4)	0.015 (2)	0.006 (3)	0.006 (2)
C19	0.051 (2)	0.054 (2)	0.113 (3)	0.0055 (19)	0.002 (2)	0.010 (2)
C20	0.066 (2)	0.042 (2)	0.082 (3)	−0.0017 (18)	0.022 (2)	0.0144 (18)
C21	0.058 (2)	0.060 (2)	0.074 (3)	−0.0027 (18)	0.027 (2)	0.0179 (19)
C22	0.050 (2)	0.054 (2)	0.064 (2)	−0.0010 (17)	0.0192 (18)	0.0095 (17)
C23	0.0398 (18)	0.0437 (19)	0.048 (2)	−0.0021 (15)	0.0099 (16)	0.0029 (15)
C24	0.0448 (18)	0.0329 (17)	0.049 (2)	−0.0007 (14)	0.0094 (16)	0.0009 (14)
C25	0.0377 (17)	0.0419 (18)	0.0472 (19)	−0.0007 (14)	0.0112 (15)	−0.0022 (15)
C26	0.053 (2)	0.051 (2)	0.066 (2)	0.0059 (17)	0.0263 (19)	−0.0033 (18)
C27	0.057 (2)	0.044 (2)	0.070 (2)	−0.0008 (16)	0.0334 (19)	0.0021 (17)
C28	0.054 (2)	0.0377 (18)	0.057 (2)	−0.0006 (15)	0.0200 (18)	−0.0022 (15)
C29	0.0383 (17)	0.0378 (18)	0.047 (2)	−0.0013 (14)	0.0105 (15)	−0.0022 (14)
C30	0.0387 (17)	0.0376 (18)	0.051 (2)	−0.0024 (14)	0.0116 (16)	−0.0017 (14)
C31	0.0387 (17)	0.0407 (18)	0.0492 (19)	0.0026 (15)	0.0102 (15)	−0.0006 (15)
C32	0.0407 (18)	0.049 (2)	0.047 (2)	−0.0007 (15)	0.0164 (16)	−0.0027 (16)
C33	0.0369 (18)	0.047 (2)	0.052 (2)	0.0052 (15)	0.0083 (16)	−0.0033 (16)
C34	0.058 (2)	0.059 (2)	0.076 (3)	0.0065 (19)	0.027 (2)	0.0014 (19)
C35	0.062 (2)	0.084 (3)	0.085 (3)	0.016 (2)	0.032 (2)	−0.003 (2)
C36	0.075 (3)	0.066 (3)	0.091 (3)	0.029 (2)	0.025 (2)	−0.009 (2)
C37	0.076 (3)	0.055 (2)	0.096 (3)	0.020 (2)	0.031 (3)	0.009 (2)
C38	0.059 (2)	0.050 (2)	0.079 (3)	0.0112 (18)	0.027 (2)	0.0061 (19)
N1	0.0507 (16)	0.0428 (16)	0.0552 (18)	0.0009 (13)	0.0153 (14)	−0.0079 (13)
N2	0.0444 (15)	0.0413 (15)	0.0538 (17)	0.0008 (13)	0.0091 (14)	−0.0011 (13)
N3	0.0468 (16)	0.0396 (16)	0.0610 (18)	0.0006 (13)	0.0083 (15)	0.0011 (13)
N4	0.0453 (16)	0.0502 (17)	0.0529 (17)	0.0029 (13)	0.0081 (14)	0.0054 (14)
N5	0.0517 (16)	0.0394 (16)	0.0678 (19)	0.0027 (13)	0.0144 (15)	0.0081 (13)
N6	0.0471 (16)	0.0431 (16)	0.0648 (19)	0.0005 (13)	0.0213 (15)	0.0005 (13)
N7	0.0453 (16)	0.0458 (16)	0.0578 (18)	0.0015 (13)	0.0171 (14)	0.0024 (13)
N8	0.0429 (15)	0.0357 (15)	0.0558 (17)	0.0044 (12)	0.0171 (14)	0.0027 (12)

### Geometric parameters (Å, °)

**Table:**

C1—N2	1.321 (4)	C20—C21	1.393 (4)
C1—C2	1.388 (4)	C20—H20	0.9300
C1—H1	0.9300	C21—C22	1.369 (4)
C2—C3	1.359 (4)	C21—H21	0.9300
C2—H2	0.9300	C22—C23	1.397 (4)
C3—C4	1.402 (4)	C22—H22	0.9300
C3—H3	0.9300	C23—C24	1.407 (4)
C4—C5	1.413 (4)	C23—C31	1.432 (4)
C4—C12	1.419 (4)	C24—N5	1.359 (3)
C5—N2	1.362 (3)	C24—C25	1.461 (4)
C5—C6	1.462 (4)	C25—N6	1.356 (3)
C6—N1	1.357 (3)	C25—C29	1.412 (4)
C6—C7	1.405 (4)	C26—N6	1.323 (4)
C7—C8	1.409 (4)	C26—C27	1.389 (4)
C7—C11	1.429 (4)	C26—H26	0.9300
C8—C9	1.357 (4)	C27—C28	1.361 (4)
C8—H8	0.9300	C27—H27	0.9300
C9—C10	1.394 (4)	C28—C29	1.394 (4)
C9—H9	0.9300	C28—H28	0.9300
C10—N1	1.317 (4)	C29—C30	1.418 (4)
C10—H10	0.9300	C30—N8	1.374 (3)
C11—C12	1.373 (4)	C30—C31	1.376 (4)
C11—N4	1.377 (3)	C31—N7	1.375 (3)
C12—N3	1.375 (3)	C32—N7	1.327 (3)
C13—N4	1.334 (3)	C32—N8	1.364 (3)
C13—N3	1.368 (4)	C32—C33	1.462 (4)
C13—C14	1.464 (4)	C33—C38	1.380 (4)
C14—C15	1.375 (4)	C33—C34	1.386 (4)
C14—C19	1.384 (4)	C34—C35	1.378 (4)
C15—C16	1.390 (5)	C34—H34	0.9300
C15—H15	0.9300	C35—C36	1.374 (5)
C16—C17	1.365 (5)	C35—H35	0.9300
C16—H16	0.9300	C36—C37	1.356 (5)
C17—C18	1.364 (5)	C36—H36	0.9300
C17—H17	0.9300	C37—C38	1.378 (4)
C18—C19	1.385 (4)	C37—H37	0.9300
C18—H18	0.9300	C38—H38	0.9300
C19—H19	0.9300	N3—H3A	0.8600
C20—N5	1.315 (4)	N8—H8A	0.8600
			
N2—C1—C2	124.0 (3)	C21—C22—C23	119.7 (3)
N2—C1—H1	118.0	C21—C22—H22	120.2
C2—C1—H1	118.0	C23—C22—H22	120.2
C3—C2—C1	118.8 (3)	C22—C23—C24	118.0 (3)
C3—C2—H2	120.6	C22—C23—C31	124.0 (3)
C1—C2—H2	120.6	C24—C23—C31	118.0 (3)
C2—C3—C4	119.4 (3)	N5—C24—C23	122.1 (3)
C2—C3—H3	120.3	N5—C24—C25	117.4 (3)
C4—C3—H3	120.3	C23—C24—C25	120.5 (3)
C3—C4—C5	118.4 (3)	N6—C25—C29	121.4 (3)
C3—C4—C12	125.2 (3)	N6—C25—C24	118.1 (3)
C5—C4—C12	116.4 (3)	C29—C25—C24	120.5 (3)
N2—C5—C4	121.1 (3)	N6—C26—C27	124.1 (3)
N2—C5—C6	118.1 (3)	N6—C26—H26	117.9
C4—C5—C6	120.7 (3)	C27—C26—H26	117.9
N1—C6—C7	122.9 (3)	C28—C27—C26	118.3 (3)
N1—C6—C5	117.0 (3)	C28—C27—H27	120.8
C7—C6—C5	120.1 (3)	C26—C27—H27	120.8
C6—C7—C8	117.6 (3)	C27—C28—C29	119.8 (3)
C6—C7—C11	118.4 (3)	C27—C28—H28	120.1
C8—C7—C11	124.0 (3)	C29—C28—H28	120.1
C9—C8—C7	119.1 (3)	C28—C29—C25	118.3 (3)
C9—C8—H8	120.4	C28—C29—C30	125.1 (3)
C7—C8—H8	120.4	C25—C29—C30	116.6 (3)
C8—C9—C10	118.9 (3)	N8—C30—C31	105.0 (3)
C8—C9—H9	120.5	N8—C30—C29	130.9 (3)
C10—C9—H9	120.5	C31—C30—C29	124.0 (3)
N1—C10—C9	124.4 (3)	N7—C31—C30	111.3 (3)
N1—C10—H10	117.8	N7—C31—C23	128.4 (3)
C9—C10—H10	117.8	C30—C31—C23	120.3 (3)
C12—C11—N4	111.4 (3)	N7—C32—N8	112.1 (3)
C12—C11—C7	120.3 (3)	N7—C32—C33	124.5 (3)
N4—C11—C7	128.3 (3)	N8—C32—C33	123.4 (3)
C11—C12—N3	105.6 (3)	C38—C33—C34	118.2 (3)
C11—C12—C4	123.9 (3)	C38—C33—C32	122.6 (3)
N3—C12—C4	130.3 (3)	C34—C33—C32	119.1 (3)
N4—C13—N3	112.4 (3)	C35—C34—C33	120.4 (3)
N4—C13—C14	123.9 (3)	C35—C34—H34	119.8
N3—C13—C14	123.8 (3)	C33—C34—H34	119.8
C15—C14—C19	118.5 (3)	C36—C35—C34	120.6 (3)
C15—C14—C13	119.8 (3)	C36—C35—H35	119.7
C19—C14—C13	121.6 (3)	C34—C35—H35	119.7
C14—C15—C16	119.8 (4)	C37—C36—C35	119.2 (3)
C14—C15—H15	120.1	C37—C36—H36	120.4
C16—C15—H15	120.1	C35—C36—H36	120.4
C17—C16—C15	121.3 (4)	C36—C37—C38	121.1 (4)
C17—C16—H16	119.4	C36—C37—H37	119.5
C15—C16—H16	119.4	C38—C37—H37	119.5
C18—C17—C16	119.4 (3)	C37—C38—C33	120.5 (3)
C18—C17—H17	120.3	C37—C38—H38	119.7
C16—C17—H17	120.3	C33—C38—H38	119.7
C17—C18—C19	120.0 (4)	C10—N1—C6	117.0 (3)
C17—C18—H18	120.0	C1—N2—C5	118.2 (3)
C19—C18—H18	120.0	C13—N3—C12	106.6 (2)
C14—C19—C18	121.1 (4)	C13—N3—H3A	126.7
C14—C19—H19	119.4	C12—N3—H3A	126.7
C18—C19—H19	119.4	C13—N4—C11	104.0 (3)
N5—C20—C21	124.5 (3)	C20—N5—C24	117.6 (3)
N5—C20—H20	117.8	C26—N6—C25	118.0 (3)
C21—C20—H20	117.8	C32—N7—C31	104.4 (2)
C22—C21—C20	118.1 (3)	C32—N8—C30	107.2 (2)
C22—C21—H21	120.9	C32—N8—H8A	126.4
C20—C21—H21	120.9	C30—N8—H8A	126.4

### Hydrogen-bond geometry (Å, °)

**Table:**

*D*—H···*A*	*D*—H	H···*A*	*D*···*A*	*D*—H···*A*
N3—H3A···N6^i^	0.86	2.09	2.932 (4)	165.
N8—H8A···N2^i^	0.86	2.12	2.948 (3)	163.

Symmetry codes: (i) −*x*+3, *y*+1/2, −*z*+5/2.
